# The Role of Lipid Profile as an Independent Predictor of Non-alcoholic Steatosis and Steatohepatitis in Morbidly Obese Patients

**DOI:** 10.3389/fcvm.2021.682352

**Published:** 2021-05-31

**Authors:** Narges Ashraf Ganjooei, Tannaz Jamialahmadi, Mohsen Nematy, Ali Jangjoo, Ladan Goshayeshi, Majid Khadem-Rezaiyan, Željko Reiner, Mona Alidadi, Alexander M. Markin, Amirhossein Sahebkar

**Affiliations:** ^1^Biotechnology Research Center, Pharmaceutical Technology Institute, Mashhad University of Medical Sciences, Mashhad, Iran; ^2^Department of Nutrition, Faculty of Medicine, Mashhad University of Medical Sciences, Mashhad, Iran; ^3^Department of Food Science and Technology, Quchan Branch, Islamic Azad University, Quchan, Iran; ^4^Faculty of Medicine, Surgical Oncology Research Center, Imam Reza Hospital, Mashhad University of Medical Sciences, Mashhad, Iran; ^5^Department of Gastroenterology and Hepatology, Faculty of Medicine, Mashhad University of Medical Sciences, Mashhad, Iran; ^6^Gastroenterology and Hepatology Research Center, Mashhad University of Medical Sciences, Mashhad, Iran; ^7^Department of Community Medicine and Public Health, Faculty of Medicine, Mashhad University of Medical Sciences, Mashhad, Iran; ^8^Department of Internal Medicine, School of Medicine, University Hospital Centre Zagreb, University of Zagreb, Zagreb, Croatia; ^9^Laboratory of Cellular and Molecular Pathology of Cardiovascular System, Institute of Human Morphology, Moscow, Russia; ^10^Applied Biomedical Research Center, Mashhad University of Medical Sciences, Mashhad, Iran; ^11^School of Pharmacy, Mashhad University of Medical Sciences, Mashhad, Iran

**Keywords:** morbid obesity, non-alcoholic fatty liver disease, two-dimensional shear wave elastography, dyslipidemia, non-alcohol based steatohepatitis

## Abstract

**Background and Aims:** Obesity is one of the major health problems worldwide. Morbid obesity (body mass index >40 kg/m^2^ or over 35 with a comorbidity) is associated, apart from other diseases, with an increased risk of non-alcoholic fatty liver disease (NAFLD). Moreover, dyslipidemia is an important comorbidity that is frequently found in NAFLD patients. The aim of this study was to analyze whether serum lipids in morbidly obese patients are associated with the spectrum of NAFLD.

**Methods:** Total serum cholesterol, LDL cholesterol, HDL cholesterol, non-HDL cholesterol, VLDL, and triglycerides were analyzed in 90 morbidly obese patients. The association of lipid profile parameters with histopathological, elastographic, and sonographic indices of NAFLD, non-alcoholic steatohepatitis (NASH), and liver fibrosis were explored.

**Results:** The mean levels of serum total cholesterol, LDL-C, and non-HDL cholesterol in patients with positive histology for liver steatosis and NASH were significantly higher than those in patients with negative histology. None of the indices showed a strong association with NAFLD, NASH, or liver fibrosis after adjustment for potential confounders.

**Conclusion:** A slight predictive value of lipid profile is not sufficiently enough to use solely as a non-invasive test in predicting NASH or liver fibrosis.

## Introduction

Non-alcoholic fatty liver disease (NAFLD)—the most common cause of liver disease—is described as the presence of hepatic fat accumulation exceeding 5% of liver weight in the absence of excessive alcohol use. It can progress to non-alcoholic steatohepatitis (NASH), cirrhosis, and even hepatocellular carcinoma (1–4). Moreover, NAFLD increases the risk of incident chronic diseases including cardiovascular disease, type 2 diabetes, and chronic kidney disease (2, 5–7). The overall worldwide NAFLD prevalence is 25.2%, but the prevalence varies between nations (8). Although about 3–30% of NAFLD patients have a relatively normal body mass index (BMI), NAFLD is strongly associated with obesity and hyperlipidemia, and it seems to be the hepatic manifestation of metabolic syndrome (4, 9–12).

Several methods, both invasive and non-invasive, have been suggested to evaluate liver fat content, NASH and fibrosis. Though liver biopsy is still regarded as the gold standard for diagnosing NAFLD, due to its various limitations, finding a safe, non-invasive, and accurate method is needed (13, 14). Dyslipidemia, which is characterized by hypertriglyceridemia, reductions in high-density lipoprotein cholesterol (HDL-c), and increase in very low-density lipoprotein (VLDL) and low-density lipoprotein cholesterol (LDL-c), is an important comorbidity that is frequently found in NAFLD patients (15, 16). Emerging data suggest that lipid profile parameters may be associated with NAFLD severity and the development of NASH and liver fibrosis (17–20).

In the present study, we performed a prospective cohort study to determine whether lipid profile components are an independent predictor of NAFLD in a morbidly obese population. Moreover, their optimal cutoff point for detecting NAFLD was also determined.

## Materials and Methods

Morbidly obese patients with BMI higher than 40 kg/m^2^ or over 35 with one or more comorbidity were recruited from the outpatient clinic between December 2016 and September 2017. Psychological assessment and medical examination was done before surgery to exclude patients with absolute contraindication to bariatric surgery. Each participant fulfilled the informed consent. Males and females who met the following criteria were included: alcohol drinking not more than 30 and 20 g/day, respectively, no consumption or just temporary consumption of hepatotoxic medications, and negative HBV and HCV antibodies. Eventually, 90 patients were selected. All procedures performed in this study were in accordance with the ethical standards of the institutional and/or national research committee and with the 1964 Helsinki Declaration and its later amendments or comparable ethical standards.

### Two-Dimensional Shear Wave Elastography

Liver stiffness was assessed by two-dimensional shear wave elastography (2D-SWE) in real time using B-mode ultrasound imaging with potential to select the region of interest. During the 2-week preoperative period, liver stiffness (2D-SWE) was measured. Aixplorer ultrasound system (Supersonic Imagine, France) and a convex broadband probe (SC6-1, 1–6 MHz) were used based on instructions provided by manufacturer. The ideal position—hold the arm completely abducted in the right dorsal decubitus—was proposed after 6-h fasting. An acceptable liver stiffness measurement was based on 10 acquisitions measured in each participant. The mean (*M*) of valid measurements in kilopascals (kPa) was considered as a result of liver stiffness evaluation for each subject. The single operator was blinded to the study data.

### Histologic Analysis of the Hepatic Tissue

Liver tissue biopsies were obtained during the bariatric procedure from the left lobe with 16-gauge Tru-cut needle. Patients with abnormal liver function tests and liver steatosis as confirmed by ultrasound or direct view during surgery were eligible for biopsy. The specimens were stained with hematoxylin–eosin–saffron, Masson's trichrome, and picrosirius red after embedding in paraffin for histologic assessment. The expert pathologist who studied the biopsy samples was also blinded to the patients' data and disease. NASH Clinical Research Network Modified Brunt methodology and NASH Activity Score (NAS) were used for staging and grading of NASH, respectively (21). Scores were given according to a scoring system based on 2D-SWE results as follows: five stages of hepatic fibrosis (scored from 0 to 4), percentage of involved portions for hepatic steatosis [scored from 0 to 3 (0, <5%; 1, 5–33%; 2, 34–66%; 3, >66%)], number of diagnosed foci in a ×20 magnitude for lobular inflammation (scored from 0 to 3; 0: none, 1: 1–2, 2: 2–4, 3: >4), and number of ballooned hepatocytes in hepatocellular ballooning (scored from 0 to 2; 0: none, 1: few, 2: many). The total sum of all the above-mentioned scores was reported individually as NAS score for each patient. Based on this, the patients were classified in two groups as follows: no NASH (0–2 points) and definite NASH (21, 22).

### Statistical Analysis

Demographic variables were described by descriptive statistics. Mean (standard deviation, SD) and median (interquartile range, IQR) were reported using parametric and non-parametric values, respectively. Spearman's rank correlation coefficient was used to determine the correlation between ordinal variables. To demonstrate the diagnostic accuracy of lipoproteins and define the optimal cutoff point, receiver operating characteristic (ROC) curves were plotted. Sensitivity, specificity, and areas under the ROC curves (AUC) for the corresponding data were also determined by DeLong's method for correlated data. SPSS (version 25) was used for statistical analysis. Subsequently, the predicted lipoprotein cutoffs were constructed, and AUC was calculated. The *p*-value for all tests, if applicable, was considered significant at the level of 5%.

## Results

### Patients' Characteristics

Ninety patients were included in the study. Their mean age was 38.5 ± 11.1 years, and the mean BMI was 45.46 ± 6.26 kg/m^2^. More than half of them (51.9%) had metabolic syndrome, 38 had no fibrosis (*F* < 1), and 52 had fibrosis (*F* ≥ 1). Severe steatosis (>66%) was detected in 8.9% patients, and NASH was found in more than half of the patients (Table 1).

**Table 1:** Patients' characteristics.

**Variable**	**Total**
Male	18 (20)
Age	38.5 ± 11.1
BMI	45.46 ± 6.26
Weight	121.34 ± 20.32
Waist circumference	133.04 ± 13.6
Height	1.62 ± 8.87
Diabetes type 2	25 (27.8)
Hypertension	23 (25.6)
Metabolic syndrome	46 (51.1)
Liver stiffness measurement (kPa)	6.1 ± 1.25
**Fibrosis stage**	
0 = no fibrosis	38 (42.2)
1 = zone 3 perivenular or pericellular fibrosis	40 (44.4)
2 = stage 1 plus portal fibrosis	8 (8.8)
3 = bridging fibrosis, focal or extensive	4 (4.4)
4 = residual pericellular fibrosis	–
**NASH status**	
No NASH (0–2)	39 (43.3)
NASH (3–8)	51 (56.7)
**Steatosis status**	
S0: <5%	39 (43.3)
S1: 5–33%	31 (34.4)
S2: 34–66%	12 (13.3)
S3: > 66%	8 (8.9)

*Data are presented as N (%) or mean ± SD*.

*BMI, body mass index; NASH, non-alcoholic steatohepatitis*.

### Lipid Profile Parameter Concentration Based on Fatty Liver Disease

A comparison of serum lipids between study groups is presented in Table 2. The mean levels of serum total cholesterol and non-HDL cholesterol in patients with positive histology for liver steatosis and NASH were significantly higher than in patients with negative histology. In patients with positive histology for liver fibrosis, steatosis, and NASH, the mean level of LDL-C was also significantly higher when compared with patients who had negative histology.

**Table 2:** The comparison of serum parameter concentration between study groups.

	**Cholesterol**	**LDL-C**	**Non-HDL cholesterol**	**HDL-C^a^**	**TGs^a^**	**VLDL^a^**
**Fibrosis status (biopsy)**						
No fibrosis	165.2 ± 41	93.1 ± 34^*^	121.3 ± 40	45 (38–47)	119 (87–167)	23.5 (19–33)^*^
Fibrosis	182.5 ± 40	106.9 ± 30	136.8 ± 41	44 (41–48)	138 (108–184)	28 (22–39)
**NASH status**						
No NASH (0–2)	164.2 ± 41^*^	92.8 ± 33^*^	120.4 ± 40^*^	45 (39–47)	121 (87–170)	24 (20–34)
NASH (3–8)	184.1 ± 40	107.6 ± 31	138.2 ± 41	44 (42–48)	138 (108–181)	28 (22-38)
**Steatosis status (biopsy)**						
No steatosis (<5%)	164.2 ± 41^*^	92.8 ± 33^*^	120.4 ± 40^*^	45 (39–47)	121 (87–170)	24 (20–34)
Steatosis (≥5%)	183.6 ± 39	107.3 ± 30	137.9 ± 41	44 (42–48)	137.5 (108–180)	28 (22–37)
**Fibrosis status (elasto)**						
No fibrosis	170.3 ± 40	95.7 ± 30	125.6 ± 38	45 (42–47)	117 (95–153)	23.5 (20–29)^*^
Fibrosis (fibrosis cutoff = 5.85 kPa)	180 ± 42	106.6 ± 33	134.3 ± 44	44 (40–48)	138 (101–190)	28.5 (21–39)
**Steatosis status (sono)**						
No steatosis (0–1)	163.5 ± 42	94.4 ± 37	117.6 ± 41	47 (41–48)	112 (86–142)^*^	22 (20–28)^*^
Steatosis (>1)	179.8 ± 40	104.2 ± 30	134.7 ± 41	44 (40–47)	137.5 (105–183)	27 (21–37)

*Values are means ± SD*.

*LDL, low-density lipoprotein; HDL, high-density lipoprotein; TG, triglyceride; VLDL, very-low density lipoprotein*.

a *Mann–Whitney test; values are median ± interquartile range*.

* *P < 0.05 between the groups*.

The median serum concentration of HDL-C was not significantly different between the groups, but based on ultrasonography, the median serum triglyceride (TG) level in patients with steatosis was significantly higher than in patients without steatosis. VLDL was also higher in patients who had been diagnosed with steatosis (ultrasonography) and fibrosis (based on histology and elastography).

### The Relationship Between Serum Lipids and Liver Status

The relationships between lipid profile and liver fibrosis, NASH, liver steatosis, liver elastography, and ultrasonography are presented in Table 3.

**Table 3:** Correlation coefficient between parameters.

**CC**	***R***	***p*-value**
**Cholesterol**		
Fibrosis (biopsy)	0.152	0.155
Steatosis (biopsy)	0.279	0.008
NASH	0.234	0.028
Elastography	0.078	0.475
Ultrasonography	0.215	0.470
**LDL**		
Fibrosis (biopsy)	0.167	0.118
Steatosis (biopsy)	0.276	0.009
NASH	0.241	0.024
Elastography	0.107	0.327
Ultrasonography	0.185	0.087
**Non-HDL cholesterol**		
Fibrosis (biopsy)	0.111	0.303
Steatosis (biopsy)	0.231	0.030
NASH	0.173	0.109
Elastography	0.092	0.402
Ultrasonography	0.218	0.045
**HDL-C**		
Fibrosis (biopsy)	0.096	0.373
Steatosis (biopsy)	0.108	0.316
NASH	0.149	0.169
Elastography	−0.126	0.250
Ultrasonography	−0.098	0.372
**TGs**		
Fibrosis (biopsy)	0.183	0.087
Steatosis (biopsy)	0.159	0.137
NASH	0.171	0.111
Elastography	0.188	0.084
Ultrasonography	0.358	0.001
**VLDL**		
Fibrosis (biopsy)	0.225	0.039
Steatosis (biopsy)	0.210	0.055
NASH	0.216	0.050
Elastography	0.266	0.016
Ultrasonography	0.335	0.002

*CC, correlation coefficient; NASH, non-alcoholic steatohepatitis; LDL, low-density lipoprotein; HDL, high-density lipoprotein; TG, triglyceride; VLDL, very-low density lipoprotein*.

As seen in Table 3, cholesterol and LDL levels were positively correlated with NASH and steatosis (biopsy). The serum levels of non-HDL cholesterol, TG, and VLDL were positively correlated with steatosis (ultrasonography) (*p* = 0.045, *p* = 0.001, and *p* = 0.002, respectively). Moreover, aside from steatosis (ultrasonography), VLDL was also positively correlated with fibrosis (biopsy) and elastography (*p* = 0.039 and *p* = 0.016, respectively).

### Diagnostic Importance of Serum Lipids in Assessing Liver Disease

**Figure 1:**
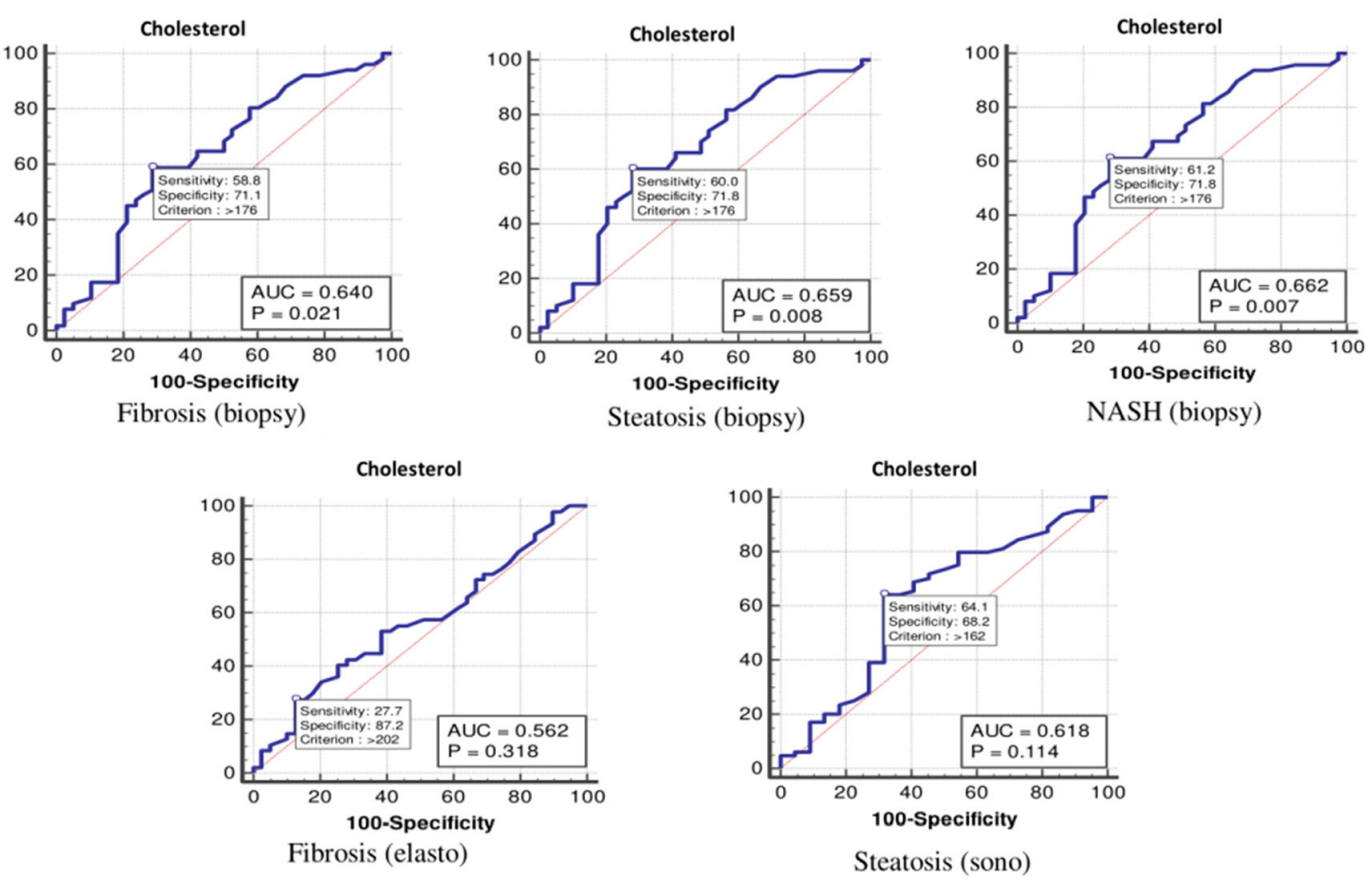
The receiver operating characteristics curve for cholesterol in the detection of liver disease.

**Figure 2:**
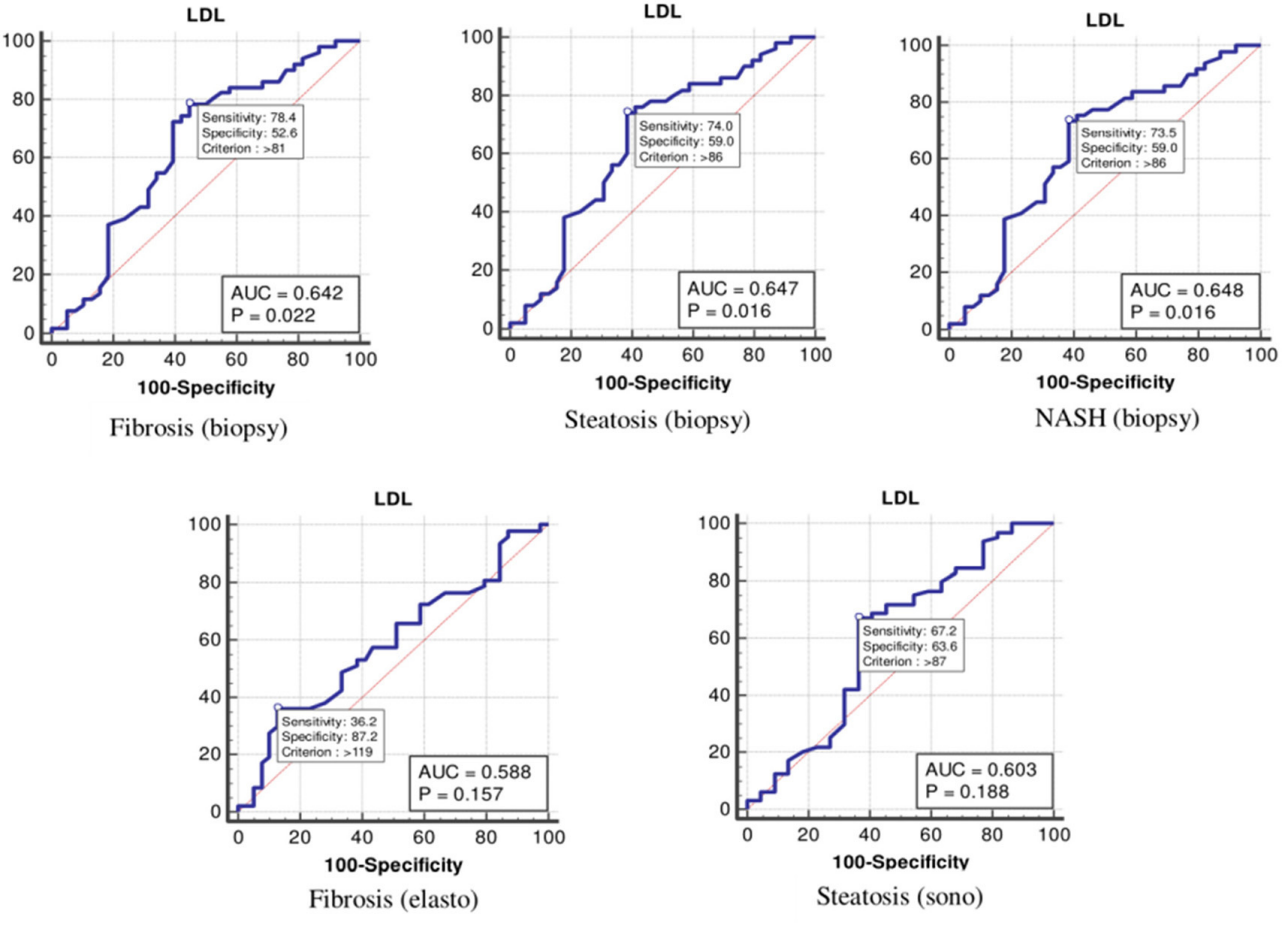
The receiver operating characteristics curve for low-density lipoprotein cholesterol in the detection of liver disease.

**Figure 3:**
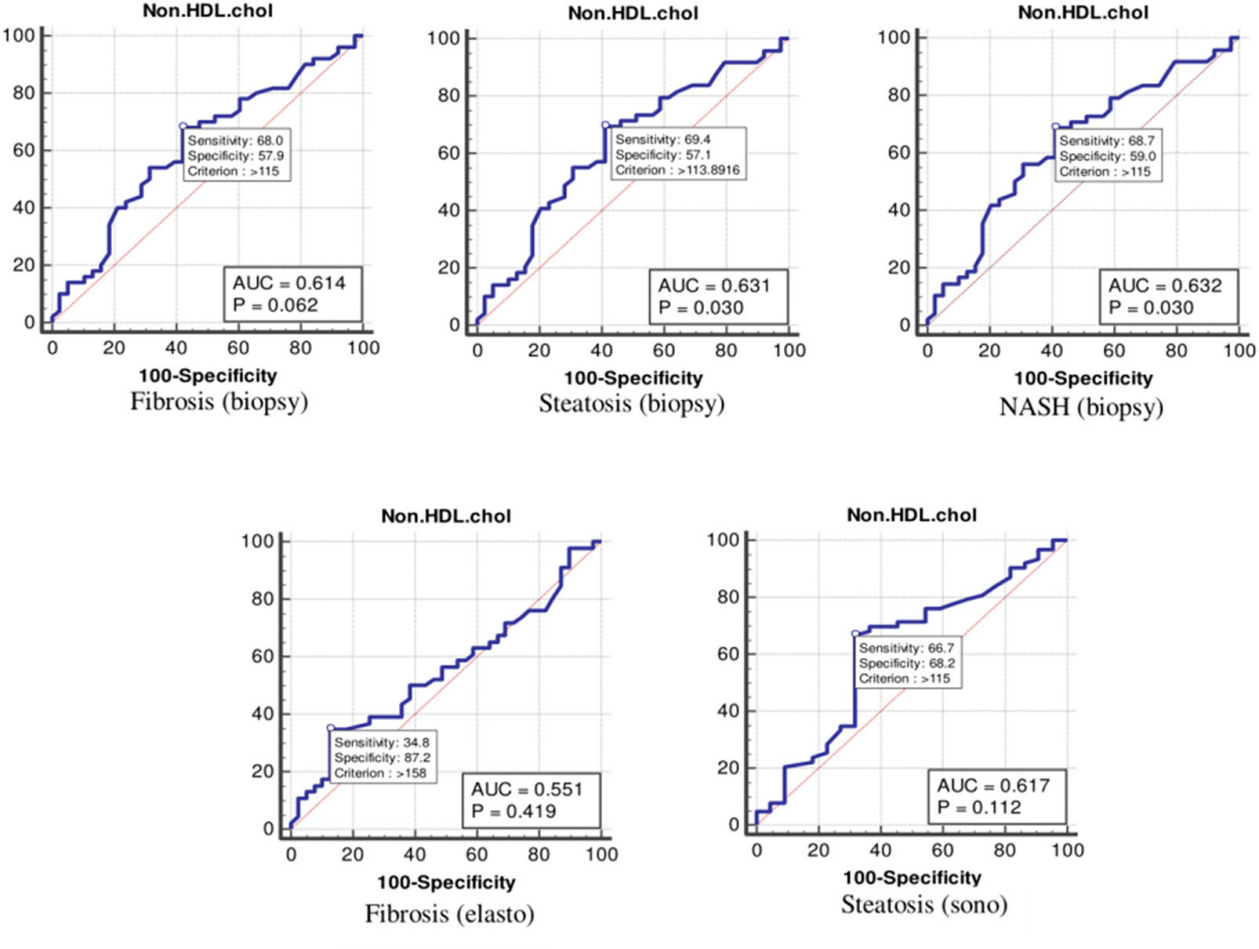
The receiver operating characteristics curve for non-high-density lipoprotein cholesterol in the detection of liver disease.

**Figure 4:**
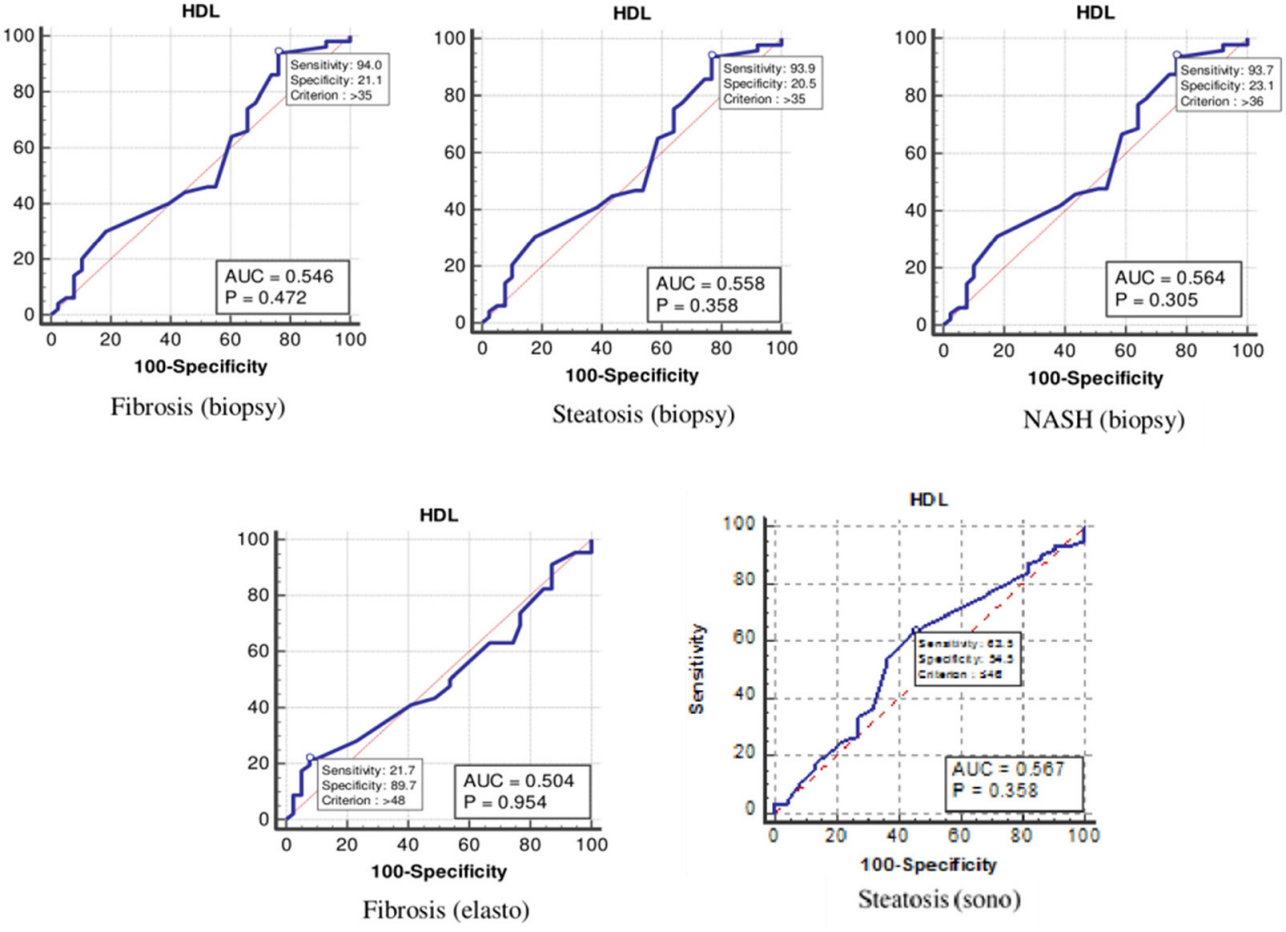
The receiver operating characteristics curve for low-density lipoprotein cholesterol in the detection of liver disease.

**Figure 5:**
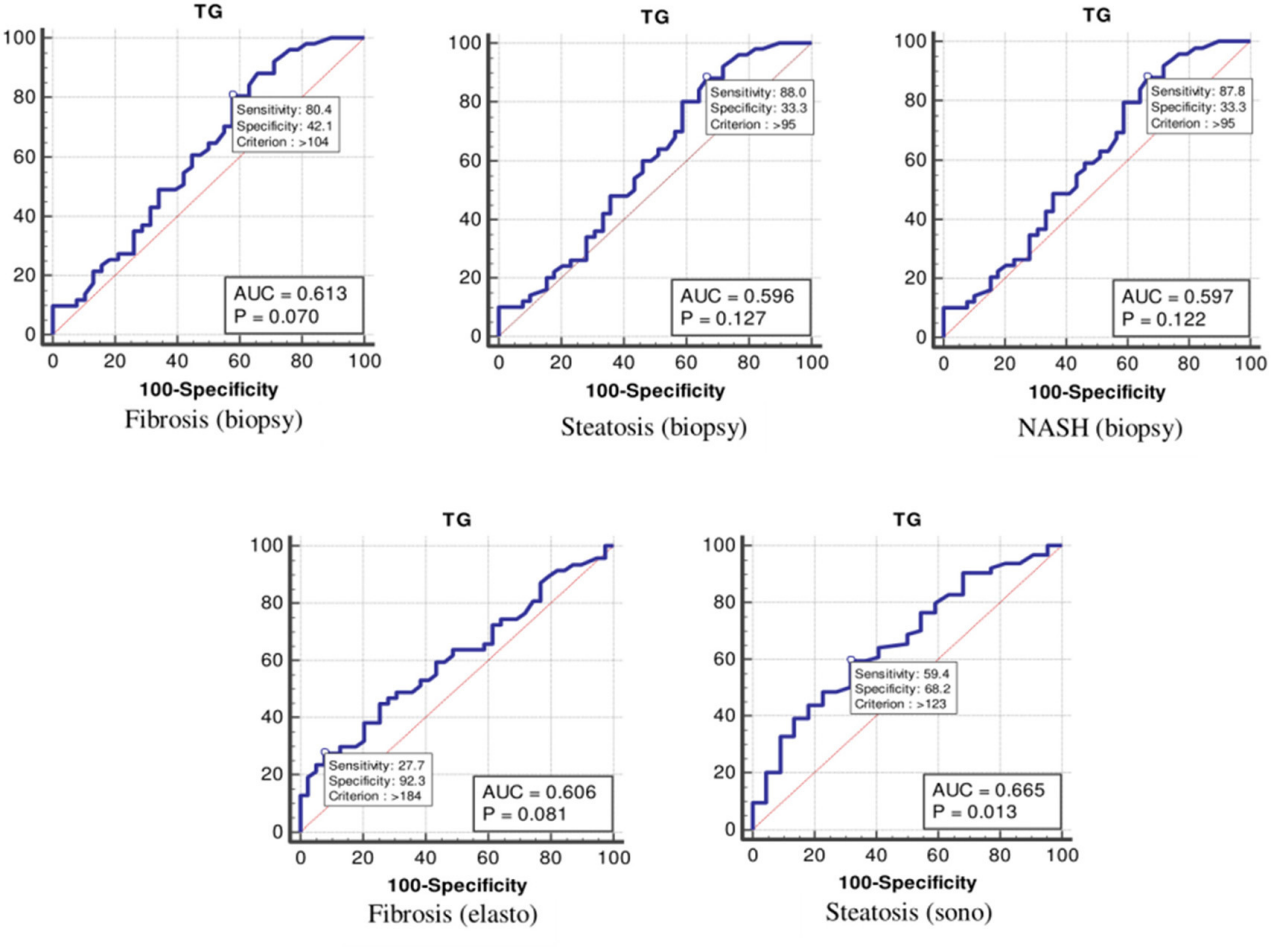
The receiver operating characteristics curve for triglycerides in the detection of liver disease.

**Figure 6:**
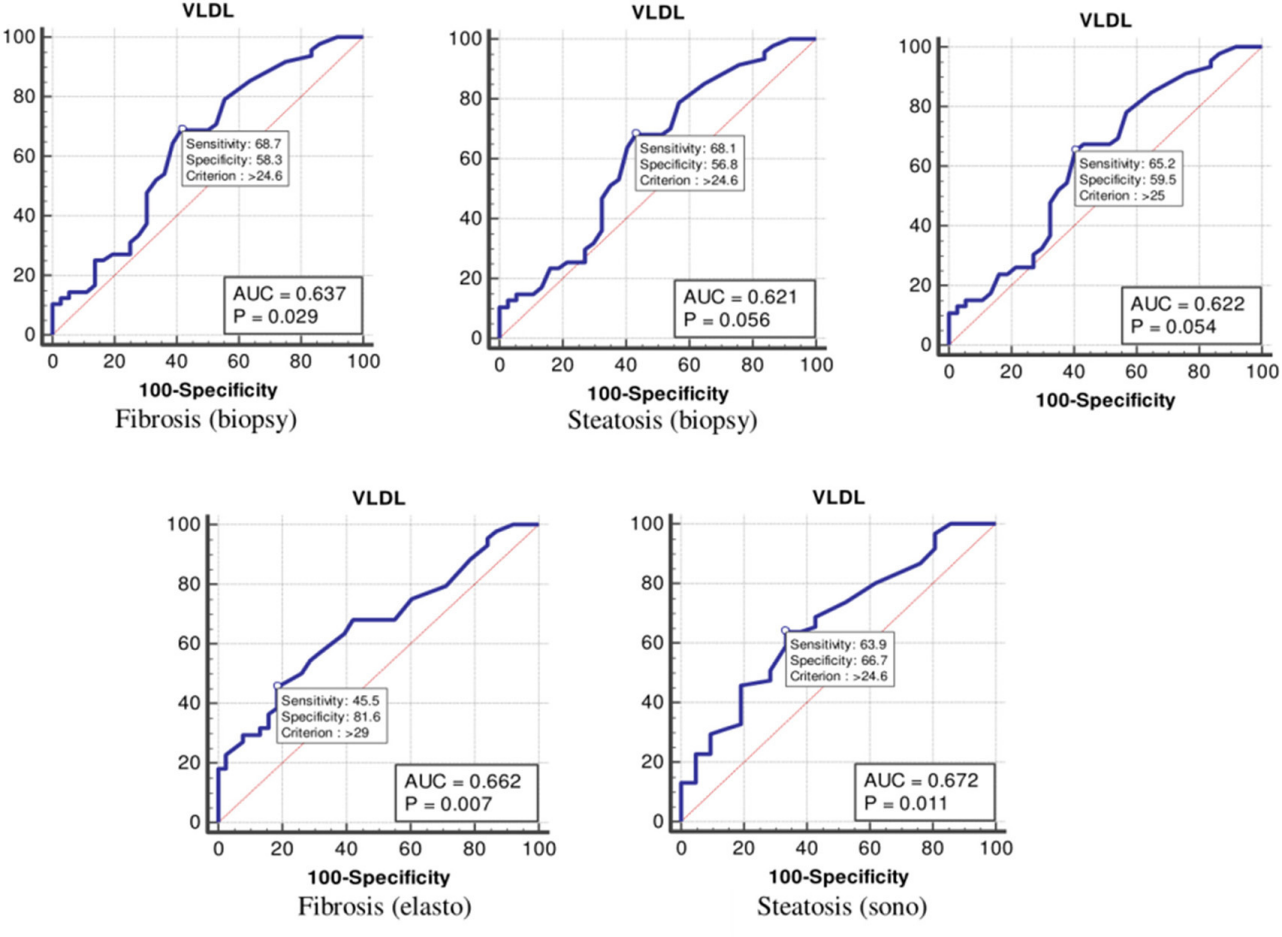
The receiver operating characteristics curve for very low-density lipoprotein in the detection of liver disease.

The values were determined using the ROC curves as optimal cutoff points. The sensitivity and specificity for each NASH CRN-modified BRUNT methodology stage are summarized in Table 4 and Figures 1–**6**.

**Table 4:** Diagnostic accuracy of serum lipids in liver disease.

**Lipid**		**AUC**	**Cutoff**	**Sens (%)**	**Spec (%)**
Total cholesterol	Fibrosis (biopsy)	0.64	176	58	71
	Steatosis (biopsy)	0.65	176	60	71
	NASH (biopsy)	0.66	176	61	71
	Fibrosis (elastography)	0.56	202	27	87
	Steatosis (ultrasonography)	0.61	162	64	68
LDL-C	Fibrosis (biopsy)	0.64	81	78	52
	Steatosis (biopsy)	0.64	86	74	59
	NASH (biopsy)	0.64	86	73	59
	Fibrosis (elastography)	0.58	87	36	87
	Steatosis (ultrasonography)	0.60	87	67	63
Non-HDL-C	Fibrosis (biopsy)	0.64	115	68	57
	Steatosis (biopsy)	0.64	113	69	57
	NASH (biopsy)	0.64	115	68	59
	Fibrosis (elastography)	0.55	158	34	87
	Steatosis (ultrasonography)	0.61	115	66	68
HDL-C	Fibrosis (biopsy)	0.54	35	94	21
	Steatosis (biopsy)	0.55	35	93	20
	NASH (biopsy)	0.56	36	93	23
	Fibrosis (elastography)	0.50	48	21	89
	Steatosis (ultrasonography)	0.56	46	63	54
TGs	Fibrosis (biopsy)	0.61	104	80	42
	Steatosis (biopsy)	0.59	95	88	33
	NASH (biopsy)	0.59	95	87	33
	Fibrosis (elastography)	0.60	184	27	92
	Steatosis (ultrasonography)	0.66	123	59	68
VLDL-C	Fibrosis (biopsy)	0.63	24	68	58
	Steatosis (biopsy)	0.62	24	68	56
	NASH (biopsy)	0.62	25	65	59
	Fibrosis (elastography)	0.66	29	45	81
	Steatosis (ultrasonography)	0.67	24	63	66

*AUC, area under the curve; Sens, sensitivity; Spec, specificity; NASH, non-alcoholic steatohepatitis*.

Based on the ROC curve, the optimal cutoff values for the total cholesterol level for detecting fibrosis (biopsy), steatosis (biopsy), NASH, fibrosis (elastography), and steatosis (ultrasonography) were 176, 176, 176, 202, and 162 mg/dl, respectively (Table 4 and Figure 1).

As shown in Table 4 and Figure 2, the optimal cutoff values for the LDL-C level for detecting fibrosis (biopsy), steatosis (biopsy), NASH, fibrosis (elastography), and steatosis (ultrasonography) were 81, 86, 86, 87, and 87 mg/dl, and the *P*-values were 0.022, 0.016, 0.016, 0.157, and 0.188, respectively.

According to the ROC curve analysis (Table 4 and Figure 3), the optimal cutoff values of non-HDL cholesterol for liver fibrosis (biopsy), liver steatosis (biopsy), NASH score, liver fibrosis (elastography), and liver steatosis (ultrasonography) were also 115 (*p* = 0.062), 113 (*p* = 0.030), 115 (*p* = 0.030), 158 (*p* = 0.419), and 115 (*p* = 0.112) mg/dl, respectively.

As Table 4 and Figure 4 show, the optimal cutoff values for the HDL-C level for liver fibrosis (biopsy), liver steatosis (biopsy), NASH score, liver fibrosis (elastography), and liver steatosis (ultrasonography) were 35, 35, 36, 48, and 46 mg/dl, respectively.

Moreover, the ROC curve (Table 4 and Figure 5) indicated that the optimal cutoff values for TGs for liver fibrosis (biopsy), liver steatosis (biopsy), NASH score, liver fibrosis (elastography), and liver steatosis (ultrasonography) were 104 (*p* = 0.070), 95 (*p* = 0.127), 95 (*p* = 0.122), 184 (*p* = 0.081), and 123 (*p* = 0.013) mg/dl, respectively.

Finally, the optimal cutoff values for the VLDL level for liver fibrosis (biopsy), liver steatosis (biopsy), NASH score, liver fibrosis (elastography), and liver steatosis (ultrasonography) were 24, 24, 25, 29, and 24 mg/dl, respectively (Table 4 and Figure 6).

### The Binary Logistic Regression Analysis Between Lipids and Study Parameters

Binary logistic regression analysis for each liver parameter was analyzed after adjusting for age, sex, waist circumference, aspartate aminotransferase, alanine aminotransferase, gamma glutamil transferase, alkaline phosphatase, lipids, and homeostatic model assessment for insulin resistance (Table 5). The binary logistic regression analysis showed that, although some serum lipids were predictive for liver histology in unadjusted models, none of them was a predictive factor in adjusted models.

**Table 5:** The binary logistic regression analysis between lipid profile and liver study parameters.

**Parameters**		**Crude model**		**Adjusted model**	
		***p***	**OR (95% CI)**	***p***	**OR (95% CI)**
Total cholesterol	Fibrosis (biopsy)	0.053	1.011 (1.000–1.022)	0.373	1.008 (0.991–1.025)
	Steatosis (biopsy)	0.030	1.012 (1.001–1.024)	0.280	1.010 (0.999–1.027)
	NASH	0.027	1.013 (1.001–1.024)	0.280	1.010 (0.999–1.027)
	Fibrosis (elastography)	0.275	1.006 (0.995–1.017)	0.784	1.002 (0.989–1.015)
	Steatosis (sono)	0.110	1.011 (0.998–1.024)	0.386	1.007 (0.992–1.022)
HDL-C	Fibrosis (biopsy)	0.379	1.027 (0.968–1.089)	0.739	1.014 (0.933–1.103)
	Steatosis (biopsy)	0.313	1.031 (0.972–1.093)	0.630	1.021 (0.938–1.111)
	NASH	0.266	1.034 (0.975–1.098)	0.630	1.021 (0.938–1.111)
	Fibrosis (elastography)	0.718	1.011 (0.953–1.073)	0.572	1.024 (0.943–1.111)
	Steatosis (sono)	0.563	0.981 (0.981–1.048)	0.759	0.985 (0.892–1.087)
LDL-C	Fibrosis (biopsy)	0.047	1.015 (1.000–1.029)	0.286	1.010 (0.991–1.030)
	Steatosis (biopsy)	0.037	1.015 (1.001–1.030)	0.251	1.011 (0.992–1.031)
	NASH	0.036	1.015 (1.001–1.030)	0.251	1.011 (0.992–1.031)
	Fibrosis (elastography)	0.120	1.011 (0.997–1.025)	0.251	1.011 (0.993–1.028)
	Steatosis (sono)	0.219	1.010 (0.994–1.027)	0.785	1.003 (0.984–1.021)
TG	Fibrosis (biopsy)	0.092	1.006 (0.999–1.014)	0.411	1.005 (0.994–1.015)
	Steatosis (biopsy)	0.120	1.006 (0.999–1.012)	0.597	1.003 (0.992–1.013)
	NASH	0.115	1.006 (0.999–1.013)	0.597	1.003 (0.992–1.013)
	Fibrosis (elastography)	0.040	1.009 (1.000–1.017)	0.203	1.008 (0.996–1.019)
	Steatosis (sono)	0.041	1.013 (1.001–1.025)	0.194	1.010 (0.995–1.025)
VLDL-C	Fibrosis (biopsy)	0.072	1.036 (0.997–1.077)	0.376	1.025 (0.970–1.084)
	Steatosis (biopsy)	0.092	1.032 (0.995–1.071)	0.532	1.017 (0.965–1.72)
	NASH	0.087	1.033 (0.995–1.071)	0.532	1.017 (0.965–1.72)
	Fibrosis (elastography)	0.013	1.065 (1.013–1.120)	0.050	1.069 (1.000–1.144)
	Steatosis (sono)	0.045	1.070 (1.001–1.144)	0.257	1.045 (0.968–1.128)
Non-HDL	Fibrosis (biopsy)	0.083	1.010 (0.999–1.021)	0.127	1.012 (0.997–1.029)
	Steatosis (biopsy)	0.051	1.011 (1.000–1.022)	0.078	1.015 (0.998–1.031)
	NASH	0.049	1.011 (1.000–1.022)	0.078	1.015 (0.998–1.031)
	Fibrosis (elastography)	0.334	1.005 (0.995–1.016)	0.725	1.002 (0.989–1.016)
	Steatosis (sono)	0.099	1.011 (0.998–1.025)	0.361	1.007 (0.992–1.023)

## Discussion

In several studies, obesity is indicated as one of the most crucial risk factors of metabolic disorders. Similarly, in this study, we have identified that about 60% of our morbidly obese population have positive histology of steatosis, NASH, or fibrosis. The results indicated that liver injury and fibrosis could be related to markers of atherogenic risk, especially VLDL serum level. There is also a link between cholesterol and LDL and NASH in this population. On the contrary, there were not any association between HDL level and degree of liver injury. Our findings identified a potential relationship between severity of liver damage and atherogenic lipid profile in morbidly obese patients with biopsy-proven NAFLD, although it was not a prominent correlation. This study principally focuses on association between liver disease severity and dyslipidemia in morbidly obese patients with NAFLD.

The recent broad use of non-invasive techniques in routine clinical practice gradually replaces biopsy due to its limitations. Since NAFLD as well as metabolic syndrome is highly prevalent in Hispanic population, NAFLD and NASH progression will be more predictable within the foreseeable future in this population (23). Due to the high visceral fat distribution in Hispanic population, the risk of NAFLD deterioration will also be increased (23). Consequently, it could be a great development to find a non-invasive screening method for such a high-risk group to reduce more adverse complications (24).

As previously reported, majority of obese patients exhibited a dyslipidemic profile (7, 25). Abnormal lipid panel is more frequent in NAFLD patients, especially with other risk factors such as obesity. It was described that VLDL levels can indicate the severity of liver injury in NAFLD patients (26, 27). Männistö et al. revealed a significant association between serum LDL and VLDL subclasses with inflammation and liver damage. Méndez-Sánchez et al. also showed that steatohepatitis and liver fibrosis are more likely to have high VLDL and LDL serum concentration than simple steatosis (24). Similarly, we investigated the relationship of VLDL cholesterol level to liver fibrosis. Both cholesterol metabolism and inflammation in the liver are potentially linked together.

Atherogenic dyslipidemia, which is described as hypertriglyceridemia, low HDL-C levels, and high LDL-C levels, is the most frequent type of lipid abnormality in NAFLD. Previous studies demonstrated that decreased serum HDL- C levels were associated with an occurrence of NAFLD, which agreed with NAFLD (28, 29). However, in this study, there was no significant association between HDL-C level and stages of NAFLD.

It is becoming increasingly evident that NAFLD is a multifactorial disease strongly related to genetic and metabolic disorders including obesity, dyslipidemia, insulin resistance, and cardiovascular diseases (30, 31). Non-invasive techniques such as in routine NAFLD screening, even in patients with risk factors, have some limitations for the assessment of NASH and liver fibrosis (32). In the same token, abnormal lipid profile is not an acceptable predictor of NAFLD in our obese patients. It has less diagnostic performance than liver biopsy as a gold-standard diagnostic modality. Accordingly, a combination of non-invasive approaches tend to have a higher accuracy in predicting liver damage than using the sole method.

It should be noted that our population was not uniform in terms of the stage of liver injury. Considering that liver damage tends to be in the lower grade, the presentation of dyslipidemia may be mild or not significant. Furthermore, there is a mix of comorbidities in this morbidly obese population, which makes it difficult to discriminate each comorbidity as a single risk factor.

## Conclusion

Although we showed that evaluating lipid profile could help in NAFLD evaluation in morbidly obese patients for disease progression, their slight predictive value is not sufficiently enough for it to be used solely as a non-invasive test in NASH or NAFLD fibrosis. Therefore, early diagnosis of NAFLD using a cost-effective diagnostic approach is needed.

## Data Availability Statement

The raw data associated with this study will be available from the corresponding author upon a reasonable request.

## Ethics Statement

All procedures performed in this study were in accordance with the ethical standards of the institutional and/or national research committee and with the 1964 Helsinki Declaration and its later amendments or comparable ethical standards. The patients/participants provided their written informed consent to participate in this study.

## Author Contributions

AS and MN conceived the study. NG, TJ, MK-R, MA, and ŽR wrote the manuscript. AS, AM, AJ, and LG revised the manuscript draft. All authors approved the final version and agreed with submission.

## Conflict of Interest

The authors declare that the research was conducted in the absence of any commercial or financial relationships that could be construed as a potential conflict of interest.

## Abbreviations

AST: aspartate aminotransferase
ALT: alanine aminotransferase
ALP: alkaline phosphatase
AUROCs: areas under the ROC curves
BMI: body mass index
GGT: gamma glutamil transferase
HOMA-IR: homeostatic model assessment for insulin resistance
HDL-c: high-density lipoprotein cholesterol
HBV: hepatitis B virus
HCV: hepatitis C virus
IQR: interquartile range
LSM: liver stiffness measurement
LSE: liver stiffness evaluation
LDL-c: low-density lipoprotein cholesterol
kPa: kilopascals
NAFLD: non-alcoholic fatty liver disease
NASH: non-alcoholic steatohepatitis
NAS: NASH Activity Score
ROC: receiver operating characteristic
TG: triglycerides
2D-SWE: two-dimensional shear wave elastography
VLDL: very low-density lipoprotein
WC: waist circumference.
